# Bleeding and Thrombotic Events in Hemodialysis Patients with Atrial Fibrillation on Anticoagulation and Antiplatelet Therapy: A 24-Month Cohort Study

**DOI:** 10.3390/medicina60111760

**Published:** 2024-10-27

**Authors:** Zorica M. Dimitrijevic, Branka P. Mitic, Danijela D. Tasic, Tamara Vrecic, Karolina Paunovic, Sonja Salinger

**Affiliations:** 1 Clinic for Nephrology, University Clinical Center Nis, 18000 Nis, Serbia; 2 Medical Faculty, University of Nis, 18000 Nis, Serbia; 3 Clinic for Cardiovascular Disesase, Univeristy Clinical Center Nis, 18000 Nis, Serbia

**Keywords:** hemodialysis, atrial fibrillation, anticoagulation therapy, bleeding, thrombosis

## Abstract

*Background and Objectives*: Patients undergoing chronic hemodialysis (HD) are predisposed to both thrombotic and bleeding complications due to the complex interplay of end-stage renal disease (ESRD), cardiovascular comorbidities, and the routine use of anticoagulant and antiplatelet therapies. This study aimed to investigate the incidence of bleeding and thrombotic events in chronic HD patients receiving anticoagulant and antiplatelet therapy, with a specific focus on those with atrial fibrillation (AF). *Materials and Methods*: A total of 224 patients, with 43 (19%) of them diagnosed with AF, were included in this single-center, observational cohort study conducted over 24 months. The cohort was divided into three groups: patients without anticoagulation, those on warfarin monotherapy, and those on combined warfarin and aspirin therapy. Bleeding events were classified as major, clinically relevant non-major bleeding (CRNMB), or minor bleeding, while thrombotic events included ischemic stroke, myocardial infarction, pulmonary embolism, and arteriovenous fistula thrombosis. *Results*: Overall, 35.7% of patients experienced a bleeding event, with major bleeding occurring in 9.4%. Patients with AF had significantly higher rates of major bleeding (18.6%) compared to those without AF (7.18%; *p* = 0.043), especially in the combined therapy group. Mortality due to bleeding was also higher in AF patients (14%). In contrast, thrombotic events occurred in 26.8% of patients, with AF patients experiencing significantly more events (48.8%) compared to non-AF patients (21.5%; *p* = 0.0006). The hazard ratio (HR) for major bleeding in patients on combined warfarin and aspirin therapy was 2.56 (*p* = 0.016), while the HR for thrombotic events was 2.34 (*p* = 0.0202). *Conclusions*: These findings highlight the increased risks of both bleeding and thrombosis in chronic HD patients with AF, particularly those on combined anticoagulation and antiplatelet therapy.

## 1. Introduction

End-stage kidney disease (ESKD) is a serious condition that necessitates continuous therapy with permanent hemodialysis (HD) procedures. While HD is a life-saving treatment, it is accompanied by various complications, especially those related to bleeding and thrombosis. These complications arise due to the complex interaction of cardiovascular comorbidities, the uremic environment, and the use of anticoagulant and antiplatelet therapy. While anticoagulation is mainly used to prevent thrombosis, it significantly increases the risk of bleeding. This bleeding tendency in patients with ESKD is multifactorial and associated with the reduced clearance of anticoagulants in patients with impaired renal function, leading to increased drug levels [1], uremic platelet dysfunction [2,3], impaired platelet–vessel wall interactions, renal anemia, and the chronic use of heparin during dialysis sessions. Establishing a balance between preventing thrombosis and mitigating the risk of bleeding represents a significant clinical challenge in treating these patients [4].

Atrial fibrillation (AF), one of the most common cardiovascular comorbidities in patients with ESKD, occurs in 3.8–27% of chronic HD patients, with the prevalence varying with age, cardiovascular health, and comorbid conditions such as hypertension and diabetes [5,6]. As AF increases the risk of thromboembolism, oral anticoagulation for stroke prevention is indicated in the vast majority of ESKD patients with AF. However, the studies on the benefit of anticoagulation in dialysis patients are inconclusive, and an excessive risk of bleeding during vitamin K antagonist treatment is observed [7,8,9]. Additionally, in some cases, antiplatelet agents are added to the treatment regimen, especially for those with ischemic heart disease. However, the combined use of anticoagulants and antiplatelet agents is associated with a further exacerbated risk of bleeding, including life-threatening episodes of intracranial and gastrointestinal bleeding [10].

As a result, clinicians encounter challenging therapeutic dilemmas in balancing the risk of thromboembolism and bleeding. The dual risks associated with anticoagulation therapy in HD patients with AF have been well documented. For example, while anticoagulant therapy is effective in reducing the incidence of cerebrovascular accidents and ischemic strokes, it can also lead to severe hemorrhagic complications, especially in patients on combination therapy [11]. This dilemma is further exacerbated by the advanced age and comorbidities of HD patients, who are often more susceptible to thrombotic and bleeding complications. Despite these risks, the optimal approach to anticoagulant treatment in this population remains unclear, with different guidelines and practices depending on the local protocols and patient characteristics. Previous studies have documented the effectiveness of anticoagulant therapy in reducing ischemic events, but this benefit is often balanced by an increased incidence of hemorrhagic complications, especially in patients receiving combination therapy [12]. Given these challenges, the optimal strategy for anticoagulation management in HD patients with AF remains a subject of ongoing debate, with variations in treatment protocols based on patient characteristics, local guidelines, and clinical judgment.

This study aimed to further investigate the incidence of bleeding and thrombosis in chronic HD patients receiving anticoagulant and antiplatelet therapy, with a particular focus on those diagnosed with AF.

## 2. Materials and Methods

This single-center, observational cohort study was conducted over a 24-month period and approved by the Ethics Committee of Nis University Hospital (approval number 39454; 1 November 2019). The study adhered to the principles outlined in the Declaration of Helsinki. All the participants provided informed consent, both oral and written, prior to their inclusion in the study.

The data on concomitant diseases and pharmacological treatments were extracted from electronic medical records, including hospital admissions, outpatient visits, and complete laboratory tests. The study team systematically reviewed the patients’ medical documentation, including discharge letters, each month to monitor for bleeding or thrombotic events.

### 2.1. Outcomes and Definitions

The primary outcome of the study was the incidence of new bleeding events.

Major bleeding was defined as by the International Society on Thrombosis and Haemostasis (ISTH), i.e., fatal bleeding and/or symptomatical bleeding in a critical area or organ, such as intracranial, intraspinal, intraocular, retroperitoneal, intraarticular, or pericardial, or intramuscular with compartment syndrome, or a drop in hemoglobin of at least 20 g/L for the last 24 h period, and/or bleeding or leading to transfusion of two or more units of whole blood or red blood cells [13].

Clinically relevant non-major bleeding (CRNMB) was defined as bleeding that did not fulfill the major bleed criteria but led to hospitalization, any intervention (medical or surgical), unscheduled visit, or change in antithrombotic therapy [14].

Minor bleeding was defined as non-life-threatening bleeding events that did not necessitate medical intervention.

Secondary outcomes included arterial and venous thrombosis events, categorized into ischemic stroke (IS), myocardial infarction (MI), pulmonary thromboembolism (PTE), and arteriovenous fistula (AVF) thrombosis. Thrombotic events (IS, MI, and PTE) were diagnosed using a combination of clinical presentation, imaging modalities (such as CT or ultrasound), and laboratory biomarkers (e.g., troponins, D-dimers) based on the standard hospital protocols for each condition. AVF thrombosis was diagnosed through clinical examination (e.g., absence of bruit or thrill) and confirmed by using Doppler ultrasound imaging.

Mortality data, including death attributable to bleeding or thrombotic events, were collected throughout the 24-month follow-up period.

### 2.2. Study Population

**Figure 1:**
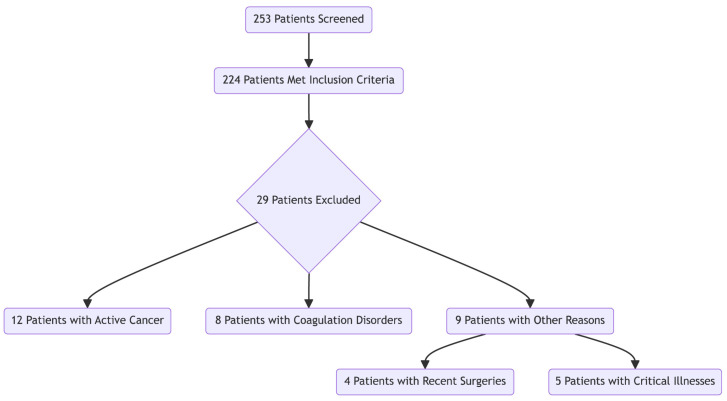
Flowchart diagram of the study cohort creation.

Patients were recruited among incident HD patients and were selected based on the predefined inclusion and exclusion criteria. All the patients receiving chronic HD at our center were eligible for inclusion if they were 18 years or older, had been on HD for at least three months, and were able to provide informed consent. The exclusion criteria included patients with active cancer, known coagulation disorders unrelated to renal disease, or contraindications to anticoagulation therapy and patients with recent surgery or critical illnesses. Of the initial 254 patients screened, 224 met the inclusion criteria after excluding those with contraindications (Figure 1).

### 2.3. Sample Size and Power Analysis

A power analysis was conducted prior to the study to determine the minimum sample size required to detect the meaningful differences in bleeding and thrombotic events between patients with atrial fibrillation (AF) and those without. Based on prior studies and the anticipated event rates of bleeding and thrombosis in HD patients, an estimated sample size of 200 patients would provide 80% power to detect a hazard ratio of 2.0 with a two-sided alpha of 0.05. With 224 patients included in the study, of whom 19% had AF, the study was adequately powered to detect the clinically significant differences in bleeding and thrombotic outcomes between the groups.

### 2.4. Anticoagulation and Antiplatelet Therapy

The patients were divided into three groups based on their anticoagulant and antiplatelet treatment regimen:

Group 1: Patients not receiving any anticoagulant or antiplatelet agents.

Group 2: Patients on warfarin monotherapy.

Group 3: Patients receiving both warfarin and aspirin for anticoagulation and antiplatelet therapy, respectively.

The treating physician guided the choice of therapy, considering each patient’s cardiovascular risk profile, comorbidities, and AF status.

Warfarin doses were adjusted based on monthly INR testing, with the goal of maintaining a therapeutic range of 2.0–3.0. All the patients underwent INR testing once a month, and dosages were modified accordingly to ensure effective anticoagulation while minimizing the risk of bleeding or thrombotic events. Although factors such as body weight and renal function were not explicitly used for dose adjustments, the INR monitoring provided a reliable measure to capture any changes related to these factors, allowing for appropriate dose modifications when necessary.

Aspirin was administered at a standard daily dose of 75–100 mg.

### 2.5. Statistical Analysis

Continuous variables were expressed as mean ± standard deviation (SD) or median with interquartile range (IQR), depending on the data distribution. Categorical variables were summarized as frequencies and percentages. The differences between the groups were analyzed using the chi-square test for categorical variables and the independent *t*-test or Mann–Whitney U test for continuous variables, as appropriate. Kaplan–Meier survival curves were generated to estimate the cumulative incidence of bleeding and thrombotic events over the 24-month follow-up. Cox proportional hazard models were used to identify the independent predictors of bleeding and thrombosis, adjusting for potential confounders such as age, gender, comorbidities, and treatment type. The assumption of proportional hazards for the Cox models was verified using Schoenfeld residuals. The global and individual tests for covariates showed no significant violations of the proportional hazards assumption (*p* > 0.05 for all tests). Log-minus-log survival plots were visually inspected, and no deviations from proportionality were observed. Therefore, the Cox proportional hazards model was considered appropriate for identifying the predictors of bleeding and thrombotic events in this cohort.

Statistical significance was set at *p* < 0.05, and all the analyses were performed using SPSS statistical software (version 26.0, IBM Corp., Armonk, NY, USA).

## 3. Results

A total of 224 patients undergoing chronic HD were included in the study. The median age of the cohort was 61 years (IQR 19–89), with 63.8% of the patients being male. The median duration of dialysis was 66.3 months (range: 7–299 months). Among the study population, 19% had atrial fibrillation (AF). The patients with AF were significantly older (median age of 64.7 years vs. 58 years for those without AF, *p* = 0.038) and had a higher prevalence of previous cardiovascular incidents, such as ischemic stroke (23.3% vs. 5%, *p* = 0.002). Among the patients with AF, 24 (55.8%) were treated with warfarin mono-therapy, while 19 (44.2%) were on a combination of warfarin and aspirin therapy. Table 1 summarizes the baseline characteristics of the study population.

### 3.1. Bleeding Events

During the follow-up period, a total of 80 patients (35.7%) experienced at least one bleeding event. Among these, 21 patients (9.4%) had major bleeding episodes, 23 patients (10.3%) had clinically relevant non-major bleeding, while 24 patients (10.7%) had minor bleeding. Twelve patients (5.3%) died due to bleeding-related complications. The data are presented in Table 2.

In the AF subgroup, bleeding events occurred in 22 patients (51.2%). The patients with AF had a notably higher percentage of any bleeding compared to those without AF, and the difference was statistically significant (*p* = 0.029). The odds ratio (OR) for any bleeding event in the patients with AF compared to those without AF was 2.21 (95% CI: 1.10–4.43), indicating that the AF patients were more than twice as likely to experience a bleeding event. Major bleeding occurred in 8 patients (18.6%) with AF and in 13 patients (7.18%) without AF (*p* = 0.043), with an OR of 2.92 (95% CI: 1.15–7.44), suggesting an almost threefold increased risk of major bleeding in the patients with AF.

During the follow-up period, major bleeding occurred more frequently in the patients on combined warfarin and aspirin therapy compared to those on warfarin monotherapy. Specifically, 18.6% of the patients on combined therapy experienced major bleeding, compared to 7.18% of the patients on warfarin alone (*p* = 0.016). The OR for major bleeding in the patients receiving combined warfarin and aspirin therapy was 2.56 (95% CI: 1.43–4.66), indicating a more than twofold increased risk of major bleeding in this group compared to that on warfarin monotherapy. For CRNMB, the OR for the patients on combined therapy was 2.34 (95% CI: 1.46–6.13, *p* = 0.0202), compared to those on warfarin alone. These results suggest that combination therapy significantly elevates the risk of both major and non-major bleeding.

A total of 12 patients (5.3%) died due to bleeding-related complications during the study period. Among those, seven patients (14%) were in the warfarin group, and five patients (3.3%) were in the non-warfarin group. The odds ratio (OR) for bleeding-related death in the warfarin group compared to the non-warfarin group was 4.72 (95% CI: 1.38–16.16, *p* = 0.016). The higher rate of fatal outcomes in the AF patients underscores the heightened bleeding risk in this subgroup, particularly among those receiving aggressive anticoagulation therapy.

### 3.2. Thrombotic Events

A total of 60 thrombotic events in 42 patients occurred during the study period, representing 26.8% of the cohort. In contrast to the increased risk of bleeding, the AF patients also faced a markedly higher risk of thrombotic events, with nearly half of the patients with AF (48.8%) experiencing at least one thrombotic event compared to 21.5% of the non-AF patients. Ischemic stroke and myocardial infarction were the most common thrombotic events, with the AF patients being significantly more likely to suffer from these complications. The rate of ischemic stroke in the AF patients (18.6%) was much higher than in those without AF (3.86%, *p* = 0.0117), reflecting the well-established link between AF and thromboembolism. Similarly, MI occurred in 11.6% of the AF patients compared to 2.8% of the non-AF patients (Table 3).

Thrombotic events contributed to a significant number of deaths in the study population. Overall, 12 patients (5.35%) died due to thrombosis-related complications. Similar to the findings for major bleeding, the patients with AF had a much higher mortality rate from thrombotic events (16.2%) compared to those without AF (2.8%, *p* = 0.0016). The higher mortality in the AF patients suggests that thrombosis is a leading cause of death in this subgroup, despite the use of anticoagulation therapy.

### 3.3. Overall Mortality

The mortality due to all causes was significantly higher in the patients with AF, with 14% of the deaths attributable to bleeding and 16.2% to thrombotic events. The hazard ratios (HR) for major bleeding and thrombotic events in the patients on combined warfarin and aspirin therapy were elevated, with an HR of 2.56 for major bleeding (*p* = 0.016) and 2.34 for clinically relevant bleeding (*p* = 0.0202) (Table 4). Warfarin monotherapy, while still associated with major bleeding, was not as strongly predictive of such events compared to the combined therapy. The patients on warfarin monotherapy had a lower incidence of thrombotic events than those on the combined therapy but still faced a notable risk of ischemic stroke (HR = 0.81, *p* = 0.11). The combined therapy group had a significantly higher rate of thrombotic events compared to the other two groups. This group had elevated hazard ratios for thrombotic events, including myocardial infarction (HR = 0.76, *p* = 0.1) and ischemic stroke (HR = 0.72, *p* = 0.683), although statistical significance was not reached in all the cases. The trend suggests that while the combined therapy may reduce some thrombotic risks, it does not fully mitigate them, and the increased risk of bleeding must also be considered.

## 4. Discussion

The findings of this study highlight a significantly enhanced risk of both bleeding and thrombotic events in chronic HD patients with AF, especially those on combined warfarin and aspirin therapy. The risk of major bleeding in the AF patients (18.6%) was notably higher compared to the non-AF patients (7.18%, *p* = 0.043), a result consistent with the current literature. Two independent studies similarly reported a significantly elevated risk of bleeding complications in dialysis patients receiving anticoagulation therapy, particularly among those treated with combined anticoagulants [15,16].

In the present study, the risk of major bleeding was more pronounced in the patients on the combined warfarin and aspirin therapy (18.6%) than in those receiving the warfarin monotherapy (7.18%, *p* = 0.016). The combined warfarin and aspirin therapy, while beneficial in reducing thrombotic risk, significantly increased the hazard ratio (HR = 2.56, *p* = 0.016) for major bleeding events compared to the warfarin monotherapy (HR = 1.14, *p* = 0.684). This finding is consistent with the results from a systematic review by Lei et al., which demonstrated that warfarin significantly increases the risks of major bleeding, including hemorrhagic stroke, in hemodialysis patients [12]. Comparable trends have been observed in other studies where the authors reported that combined anticoagulant and antiplatelet therapy was associated with a higher risk of major bleeding events in patients with chronic kidney disease (CKD), which aligns with the findings of this study [17,18]. Additionally, Daimon et al. found that bleeding risks were significantly elevated in hemodialysis patients using warfarin and aspirin, with a bleeding event rate of 9.81 per 10,000 patient-days for warfarin and 7.37 for aspirin [19].

The increased risk of thrombotic events in AF patients, particularly ischemic stroke and myocardial infarction, was another key finding of our study. Nearly half of the AF patients (48.8%) experienced a thrombotic event; 18.6% of them suffered from ischemic stroke compared to 3.86% in the non-AF patients.

This is concordant with the well-established association between AF and thromboembolic events in HD patients. Olesen et al. also found a marked increase in ischemic stroke risk in HD patients with AF, stressing the vulnerability of this patient population [20]. Our results further emphasize the potential dangers when aspirin is added to the treatment regimen.

On the other hand, the rate of myocardial infarction was remarkably higher in the AF patients (11.6%) compared to those without AF (2.8%, *p* = 0.034). Earlier research supports this finding, noting that AF is a potent risk factor for cardiovascular events, including MI, in CKD and dialysis patients [21]. 

Overall, the thrombotic event-related mortality among the AF patients (16.2%) was alarmingly high, echoing the results of the recent research reporting correspondingly high mortality rates associated with thrombotic complications in this population [22].

In contrast to other reports that suggested that warfarin monotherapy could effectively reduce the thrombotic risk without a disproportionate increase in bleeding [23,24], our findings indicate that warfarin monotherapy still carries a substantial bleeding risk. However, the bleeding risk was comparatively lower than with combined therapy.

Interestingly, despite the elevated risk of thrombotic events in the patients on combined warfarin and aspirin therapy, the risk reduction was not statistically significant in some cases, suggesting that while combined therapy may pose reduced thrombotic risks, it exposes one to significant bleeding risks. This finding is consistent with the results from prior studies which reported similar trends in anticoagulated CKD populations [25,26].

Our findings significantly impact the anticoagulation management in HD patients, especially those with AF. The study shows that although anticoagulant therapy, particularly warfarin in combination with aspirin, may provide some protection against thrombotic events, it is also associated with a high rate of bleeding complications, including major bleeding and bleeding-related mortality. This is remarkably relevant for patients with a history of cerebrovascular incidents, who showed an even higher bleeding risk when treated with the combined therapy. The increased risk of these events in this population reaffirms the need for meticulous patient evaluation and individualization of antithrombotic therapy.

Given these findings, clinicians need to carefully assess the risk–benefit ratio for anticoagulation among HD patients. Current recommendations for AF anticoagulation may underestimate the potential bleeding risk in HD patients, highlighting the need for more tailored guidelines. For instance, a critical area for future research is the development of more refined risk stratification tools to identify HD patients who are most likely to benefit from anticoagulation while minimizing the adverse outcomes. The current risk models, such as the CHA_2_DS_2_-VASc and HAS-BLED scores, may not fully capture the unique bleeding and thrombotic risks in patients with ESKD, highlighting the need for tailored scoring systems that better reflect the clinical complexness of this population. Given these risks, recent studies have highlighted the potential alternatives to warfarin, such as Direct Oral Anticoagulants (DOACs), which may provide a safer profile for patients with CKD. For example, the IRIS registry demonstrated the efficacy of rivaroxaban in reducing thromboembolic risk with potentially fewer bleeding complications in patients with CKD [27]. Additionally, the REGUEIFA registry, a multicenter study comparing vitamin K antagonists (such as warfarin) with DOACs, found that patients on DOACs reported higher satisfaction and a better health-related quality of life, as well as a lower incidence of adverse events, particularly bleeding [28].

A recent systematic review and meta-analysis conducted by de Lucena et al. [29], compared the efficacy and safety of DOACs with vitamin K antagonists in patients with AF on chronic hemodialysis. Their findings indicated that DOACs, particularly apixaban, were associated with a reduced risk of major bleeding compared to warfarin, without a significant increase in thromboembolic events. The study also suggested that DOACs offer a favorable safety profile in this high-risk population despite the concerns about their use in patients with advanced CKD due to the altered pharmacokinetics.

These findings support the growing body of literature suggesting that DOACs could be a viable alternative to warfarin in patients with CKD and AF, particularly those at high risk of bleeding. However, the use of DOACs in ESKD remains somewhat controversial, as not all DOACs have been extensively studied in this population.

Further research is needed to solidify the role of DOACs in this population, but the current evidence suggests that they may help balance the prevention of thrombotic events with the reduction in bleeding risk, a major challenge in managing AF in CKD patients.

Finally, further research is needed to explore the impact of combination therapy with anticoagulants and antiplatelet agents. Although our findings suggest an increased bleeding risk in patients on warfarin and aspirin, it is unclear whether this combination provides sufficient thrombotic protection to justify the higher risk of bleeding. Prospective studies comparing different anticoagulation strategies, including monotherapy versus combination therapy, are essential to guide more effective clinical decision making in this vulnerable group.

### Study Limitations

There were some limitations to our study that should be acknowledged. First, this was a single-center study, and the results may not be generalizable to all HD populations. Additionally, while we were able to identify the associations between anticoagulation therapy and bleeding/thrombotic outcomes, the observational design of the study precludes the ability to infer causality. Randomized controlled trials are needed to further elucidate the optimal approach to anticoagulation in HD patients with AF and to determine whether alternative therapies, such as DOACs or individualized dosing regimens, could reduce the risk of bleeding while maintaining the thromboembolic protection.

## 5. Conclusions

This study reinforces the complex relationship between anticoagulation, bleeding, and thrombosis in HD patients with AF. As previously shown, patients receiving combined warfarin and aspirin therapy encounter a significantly higher risk of bleeding, while warfarin monotherapy, though less risky, still carries substantial bleeding and thrombotic risks. Clinicians must weigh these risks carefully when considering the therapeutic strategies for HD patients, especially those with AF. Future research should focus on identifying the subgroups of patients that benefit most from anticoagulant and antiplatelet therapy while minimizing the adverse outcomes, as well as exploring alternative anticoagulation strategies that offer a more favorable risk profile in this high-risk population.

## Figures and Tables

**Table 1:** Baseline characteristics of the study population.

	All Patients	Without AF	With AF			
			Total	Warfarin + Aspirin	Warfarin	*p*-Value
Total number of patients	224	181	43 (19%)	19 (44.2%)	24 (55.8%)	
Men (%)	143 (63.8%)	100 (55.2%)	30 (69.7%)	9 (20.9%)	21 (48.4)	0.036
Age, median (IQR)	61 (19–89.0)	58.0 (19–77)	64.7 (46–89.0)	70.0 (51.0–89.0)	68.3 (43.5–76.0)	0.038
Duration of dialysis, months	66.3 (5–299)	46.3(5–173)	60.3 (89–221.0)	42.6 (25.6–202)	58.3 (31.7–198.0)	0.036
Comorbidities, *n* (%)						
Previous CVI	20 (8.9%)	10 (5%)	10 (23.3%)	2 (4.6%)	8 (18.6%)	0.002
Ischemic heart disease	33 (12.4%)	19 (10.%)	14 (32%)	6 (13.9%)	8 (18.6%)	0.71
HTA (Hypertension)	185 (69.3%)	173 (95.5%)	12 (27.9%)	5 (11.6%)	7 (16.2%)	0.75
Heart failure	43(19.9%)	37 (20.4%)	7 (16.2%)	4 (9.3%)	3 (6.9%)	0.36
Diabetes	68 (30.3%)	35 (51.4%)	33 (48.55)	20 (60.6%)	13 (39.4%)	0.022
Blood chemistry						
Hemoglobin g/L	103.2 + 11.5	102.1 ± 11.4	103.2 ± 11.1	101.3 + 10.1	102.1 + 10.2	0.9
White blood cells count, ×10^9^/L	6.2 + 2.4	6.1 ± 2.1	6.3 ± 1.7	7.1 + 2.5	6.4 + 1.1	0.9
Platelet count, ×10^9^/L	164.3 + 68.2	197.1 ± 50.2	170.5 ± 41.1	146.4 + 61.4	131.0 + 40.9	0.63
INR (PT)	1.6 + 0.4	1.3 ± 0.2	1.5.0 ± 0.1	2.0 + 0.4	2.4 + 0.1	0.09
aPTT (seconds)	33.6 + 13.7	32.2 ± 11.3	31.8 ± 13.1	33.3 + 4.6	38.8 + 9.4	0.68
Fibrinogen (g/L)	5.0 + 1.2	4.2 ± 1.2	5.2 ± 1.4	5.6 + 1.4	5.4 + 1.2	0.88
Medications						
RAAS blockers	135 (74.5%)	100 (55.2%)	35 (81.3%)	10 (23.3%)	24 (55.8%)	0.07
Calcium blockers	106 (58.5%)	84 (46.4%)	22 (51.1%)	10 (23.3%)	12 (27.9%)	0.06
Statins	31 (17.1%)	15 (8.2%)	16 (37.2%)	8 (18.6%)	8 (18.6%)	0.61

Abbreviations: AF—atrial fibrillation; CVI—cerebrovascular insult; RAAS—renin-angiotensin-aldosterone.

**Table 2:** Bleeding events by patient subgroup.

Bleeding Outcome	All the Patients (*n* = 224)	Without AF (*n* = 181)	With AF (*n* = 43)	*p* Value
Any bleeding	80 (35.7%)	58 (32.4%)	22(51.2%)	0.029
• Major bleeding	21 (9.4%)	13 (7.18%)	8 (18.6%)	0.043
• Clinically relevant bleeding	23 (10.3%)	20 (11.4%)	3 (7.0%)	0.61
• Minor bleeding	24 (10.7%)	19 (10.5%)	5 (11.6%)	1
Death due to bleeding	12 (5.3%)	6 (3.3%)	6 (14%)	0.016

**Table 3:** Thrombotic events by patient subgroup.

Thrombotic Outcome	All the Patients (*n* = 224)	Without AF (*n* = 181)	With AF (*n* = 43)	*p* Value
Any thrombotic event	60 (26.8%)	39 (21.5%)	21 (48.8%)	0.0006
• Ischemic stroke	15 (6.7%)	7 (3.86%)	8(18.6%)	0.0117
• AVF thrombosis	28 (12.5%)	21 (11.6%)	7(16.2%)	0.563
• Myocardial infarction	10 (4.5%)	5 (2.8%)	5 (11.6%)	0.034
• PTE	7 (3.5)	6(3.3)	1 (2.32)	1
Death due to thrombosis	12 (5.35%)	5 (2.8%)	7 (16.2%)	0.0016

Abbreviations: AVF—arteriovenous fistula; PTE—pulmonary thromboembolism.

**Table 4:** Hazard ratios for thrombotic and bleeding events in the patients on warfarin and the combined warfarin–aspirin therapy.

	Warfarin		Warfarin + Aspirin	
	HR (95% CI)	*p*-Value	HR (95% CI)	*p*-Value
Thrombotic event				
• Myocardial infarction	081 (0.52–0.89)	0.11	0.76 (0.67–1.28)	0.1
• PTE	0.86 (0.75–1.45)	0.361	0.88 (0.62–1.13)	0.378
• Ischemic stroke	0.77 (0.41–1.67)	0.479	0.72 (0.05–1.63)	0.683
Bleeding				
• Major bleeding	1.14 (0.92–3.12)	0.684	2.56 (1.43–4.66)	0.016
• Clinically relevant bleeding	1.31(1.11–2.81)	0.254	2.34 (1.46–6.13)	0.0202
• Minor bleeding	0.91 (0.76–1.60)	0.62	1.01 (0.91–1.28)	0.90
All cause deaths	1.79 (1.45–3.7)	0.0148	1.98 (1.68–5.12)	0.0163

Abbreviations: PTE—pulmonary thromboembolism.

## Data Availability

The data that support the findings of this study are available from the corresponding author upon reasonable request.
